# Anthropometric study of the caucasian nose in the city of Curitiba: relevance of population evaluation^☆^^☆☆^

**DOI:** 10.1016/j.bjorl.2017.06.004

**Published:** 2017-07-03

**Authors:** Annelyse Cristine Ballin, Bettina Carvalho, José Eduardo Lutaif Dolci, Renata Becker, Cezar Berger, Marcos Mocellin

**Affiliations:** a Faculdade de Ciências Médicas da Santa Casa de São Paulo, Pós-Graduação em Pesquisa em Cirurgia pelo Conselho Nacional de Desenvolvimento Científico e Tecnológico (CNPq), São Paulo, SP, Brazil; b Universidade Federal do Paraná (UFPR), Hospital das Clínicas (HC), Pós-Graduação em Saúde da Criança e do Adolescente pelo Conselho Nacional de Desenvolvimento Científico e Tecnológico (CNPq), Curitiba, PR, Brazil; c Faculdade de Ciências Médicas da Santa Casa de São Paulo, Curso de Medicina, São Paulo, SP, Brazil; d Instituto Paranaense de Otorrinolaringologia, Curitiba, PR, Brazil; e Universidade Federal do Paraná (UFPR), Hospital das Clínicas (HC), Departamento de Otorrinolaringologia, Curitiba, PR, Brazil

**Keywords:** Rhinoplasty, Anthropometry, Nose esthetics, Nose measurements, Facial plastic surgery, Rinoplastia, Antropometria, Estética nasal, Medidas nasais, Plástica facial

## Abstract

**Introduction:**

Norms and patterns of nasal esthetics are essential for an adequate preoperative evaluation and surgical programming. The esthetic nasal patterns used are a blend of artistic beauty ideals and tracings in models and celebrities. Because they do not consider population measures, they vary according to the period, and allow a discrepancy between the surgeon's preference and the patient's real desire for rhinoplasty. Not all populations wish to obtain an esthetic result according to these values, but prefer a natural result, that is, one with some of the nasal characteristics of the population to which they belong to. The Brazilian population lacks population studies to evaluate its nose measurements.

**Objective:**

(1) To evaluate the anthropometric measures of Caucasian noses of people living in the city of Curitiba (state of Paraná), and to compare them to the ideal esthetic pattern of the literature; (2) To compare them between genders.

**Methods:**

This is a prospective cohort study involving 100 Caucasian volunteers at a tertiary hospital in Southern Brazil. Through the frontal and lateral view photos, intercanthal distance, alar distance, nasal dorsum length, nasofrontal angle, nasolabial angle, and nasal tip projection (Goode's method) were obtained. A statistical analysis was performed to compare the measures obtained between genders and with the ideal patterns.

**Results:**

Comparing the results obtained with those predicted by the esthetic ideals, the sample presented: similar nasolabial angle (*p* = 0.07), alar width greater than intercanthal distance (*p* < 0.001), higher nasal tip projection (*p* < 0.001), larger width–length ratio (*p* < 0.001), and more obtuse nasofrontal angle (*p* < 0.001). The nasofrontal angle (*p* = 0.0008) and the tip projection (*p* = 0.032) were statistically different between the genders. Men had a smaller nasofrontal angle, and a larger Goode's ratio.

**Conclusion:**

Except for the nasolabial angle, the measures obtained in the population sample differed from the published esthetic ideals. Comparing the genders, men had a sharper nasofrontal angle, and higher tip projection than women.

## Resumo

**Introdução:**

Normas e padrões de estética nasal são essenciais para uma adequada avaliação pré-operatória e programação cirúrgica. Os padrões estéticos nasais usados são uma mistura dos ideais artísticos de beleza e traçados em modelos e celebridades. Por não considerar medidas populacionais, variam conforme o período e permitem uma discrepância entre o desejo do cirurgião e o real desejo do paciente com a rinoplastia. Nem todas as populações desejam obter um resultado estético conforme esses valores, mas um resultado natural, ou seja, com algumas das características nasais da população a que pertencem. A população brasileira carece de estudos populacionais que avaliam as suas medidas nasais.

**Objetivo:**

1) Avaliar as médidas antropométricas de narizes caucasianos da cidade de Curitiba (Paraná) e compará-los com o padrão estético ideal da literatura; 2) Compará-los entre os sexos.

**Método:**

Estudo prospectivo, coorte, envolvendo 100 voluntários caucasianos em um Hospital terciário no Sul do Brasil. Através de fotografias na vista frontal e lateral, foram obtidas: distância intercantal, distância alar, comprimento do dorso nasal, ângulo nasofrontal, ângulo nasolabial e projeção da ponta nasal (método do Goode). Análise estatística foi realizada para comparar as medidas obtidas: entre os gêneros e com os padrões ideais.

**Resultados:**

Comparando os resultados obtidos com o apregoado pelos ideais estéticos, a amostra apresentou: ângulo nasolabial similar (*p* = 0,07), largura alar maior que distância intercantal (*p* < 0,001), maior projeção da ponta nasal (*p* < 0,001), relação largura-comprimento maior (*p* < 0,001) e ângulo nasofrontal mais obtuso (*p* < 0,001). Diferiram estatisticamente entre os sexos o ângulo nasofrontal (*p* = 0,0008) e a projeção da ponta (*p* = 0,032). Homens apresentaram o ângulo nasofrontal menor e a razão de Goode maior.

**Conclusão:**

Com exceção do ângulo nasolabial, as medidas obtidas na amostra populacional diferiram dos ideais estéticos publicados. Comparando os sexos, homens apresentaram um ângulo nasofrontal mais agudo e uma projeção da ponta maior que as mulheres.

## Introduction

Craniofacial anthropometry began when anthropologists measured human skulls to categorize and classify them into races. It was then discovered that the nasal index was the best index for distinguishing the different human ethnicities.^1^

The initial clinical application of craniofacial measurements occurred in cases of congenital alterations and after disfiguring facial traumas, situations in which the surgeon needed to know the standard measures, with the anthropometric studies, based on the general population, serving as an excellent basis.^2^

Since then, the development of facial morphology measures has taken place along with the development of facial plastic surgery, because facial anthropometric measures, considered as facial esthetics standards, provide objective information for an adequate preoperative evaluation and surgical programming.

The “ideal” facial measures are present in numerous books and articles about facial plastic surgery. However, there is rarely an attempt to defend its validity with studies.^3^ The patterns of facial esthetics are defined through a mix between the artistic ideas of beauty and modern trace in models and celebrities of the period.

Among the artistic ideals of beauty, the neoclassical canons stand out, with those of the facial thirds being the most popular of them.^4^ Defined by Renaissance artists, the canons established the concept of a beautiful face, and it is currently still used by plastic surgeons.^5^, ^6^ Some authors have tested the validity of neoclassical canons, and have shown that the canons do not represent average facial proportions of the population. In addition, they suggested that their prescription as ideal facial proportions should be tested.^7^, ^8^, ^9^ Later, other authors also found no similarity between facial canons and the target population.^10^, ^11^, ^12^

In a study comparing the facial proportions defined by the canons with 50 Italian models, the authors concluded that the canons seem to have changed over the centuries. In particular, they noted a relative reduction in nasal dimensions, and an increase in the width of the eyes and mouth.^13^

Currently, the parameters used in facial plastic surgery are predominantly based on the work of Powell and Humphreys, crystallized in a single book, entitled “Proportions of the Aesthetic Face”.^6^, ^14^ The authors defined an average of esthetic ideal nasal values, established through models, celebrities and patients. In addition, the authors emphasized that, because the concept of beauty is well established by the media, the values obtained for the ideal nose were obtained primarily from models of their time.^15^

Esthetic nasal ideals vary according to the historical period. For example, the ideal nasal profile of the 1960s (low and short nasal dorsum, called a “rampant” nose) has changed to a currently higher and more straight nose.^16^ This change occurred not only because the models in the current media have higher and straight noses, but also because of the understanding that a higher nose allows better breathing by maintaining greater airflow.^17^ In addition, it shows that the surgeon cannot be based on nasal aspects influenced by the current time.

Rhinoplasty aims to create an esthetically pleasing nose to the patient, without compromising nasal function. To do so, it is necessary that the surgeon has deep knowledge of both physiology and nasal esthetics. The major problem is that current nasal esthetics patterns are defined in Caucasian and American models, not in local population studies. That is, they do not consider population measures nor demographic and cultural aspects of the patient, considered relevant to achieve greater satisfaction with rhinoplasty.^18^, ^19^

Although the standard nasal measurements were based on Caucasians, not all populations wish to achieve an esthetic result according to these values. For example, Asian women prefer a nose with a more obtuse nasofrontal angle, a rounder nasal tip and a smaller projection of the nasal tip,^5^ because these characteristics resemble those observed in that population, making rhinoplasty outcomes more natural.

To better define norms and standards of nasal esthetics in the Brazilian population, population studies are necessary. The Brazilian population lacks population studies that evaluate its nasal measurements. There is great difference in the nasal aspects observed in different regions of Brazil,^20^, ^21^ Beyond that, the esthetic nasal patterns are based on Caucasians. Consequently, this study considered a Brazilian population sample that should be more similar to the esthetic standards presented in the literature. Unlike the North of Brazil, where only 22.9% of the population is white, 78.7% of the population of southern Brazil is Caucasian. The city of Curitiba was colonized by many Italian, Ukrainian, Polish and German immigrants, being composed of 76.3% of Caucasians.^22^ Therefore, Curitiba is favorable to be compared to what is reported in the literature.

## Objectives

This study's objectives are:

1. To evaluate the anthropometric measurements of Caucasian noses of inhabitants of the city of Curitiba (PR) and to compare them with the ideal nasal esthetic standard described by Powell and Humphreys.^14^
2. To compare them between genders.

## Methods

This is a prospective cohort study, carried out through a protocol and photographs, of volunteers recruited among the students of the Medical School and professionals related to the health area at Hospital de Clínicas of the Universidade Federal do Paraná (UFPR). The research project was approved by the Ethics Committee of the Institution (CAAE: 43030415.7.0000.0096).

Exclusion criteria were: age lower than 18 years, and older than 55 years, previous history of trauma, history of nasal or facial surgery, and non-Caucasians, in order to minimize ethnic variations.

All photographs were taken by the same researcher, with the same camera and the same standardization, a Sony Cyber-shot DSC-W125 7.2 Megapixel camera with fixed zoom size of 6.0 at a distance of 1.5 m between the camera and the volunteer to give uniformity for the scale and measurements. The incidences were anteroposterior and right profile.

The photos obtained underwent an analysis of the nose parameters measurements, through the software Adobe Photoshop CS3 (Adobe System, Inc, San Jose, CA, USA). Photoshop allows measuring the distance between 2 selected points and the angle between 2 lines.

**Figure 1:**
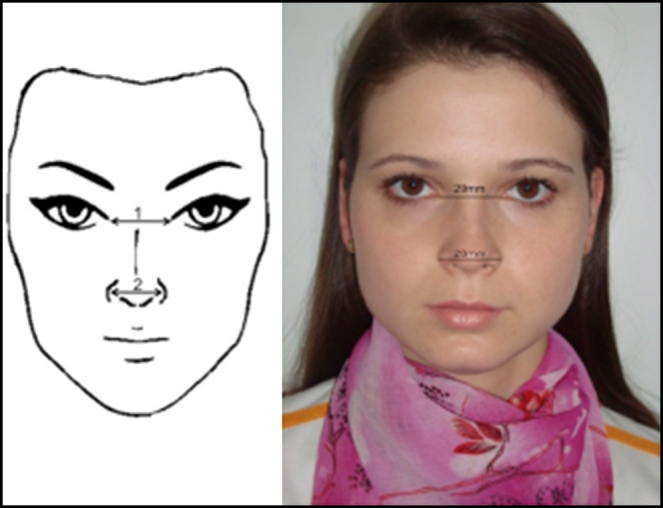
Frontal view: intercanthal distance (1) and alar distance (2). Figure on the left: schematic model. Figure on the right: one of the volunteers in the study.
- *On the lateral view (right profile) (*Fig. 2*)*:
  3. Length of the nose dorsum;
  4. Nasolabial angle (NLA);
  5. Nose tip projection (through Goode's method – Fig. 3);

The following measures were evaluated:

- *On frontal view (anteroposterior) (*Fig. 1*)*:
  1. Intercanthal distance;
  2. Alar width distance.

**Figure 3:**
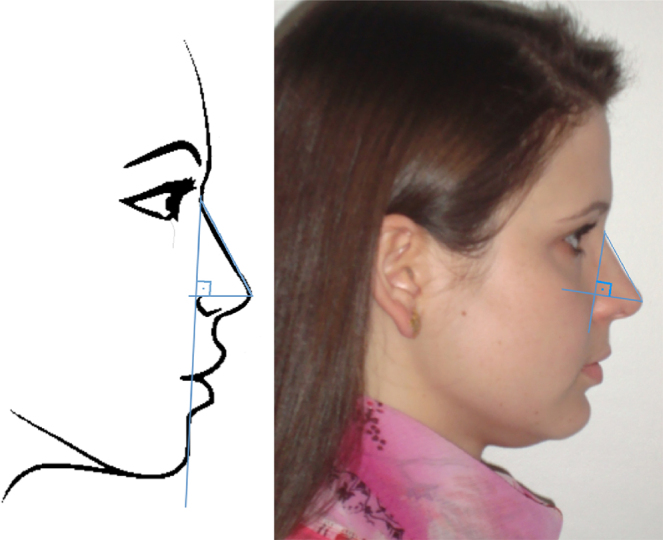
Lateral view: projection of the nasal tip, Goode's method. To calculate Goode's ratio, first a vertical line is drawn from the nasion to the alar sulcus. Then, a horizontal line is drawn to the pronasion, perpendicular to this line. The distance from the nasion to the pronasion is then measured. Goode's Ratio is obtained by dividing the alar point to the pronasion by the nasion-pronasion distance. Figure on the left: schematic model. Figure on the right: one of the volunteers in the study.
  6. Nasofrontal angle (NFA).

**Figure 2:**
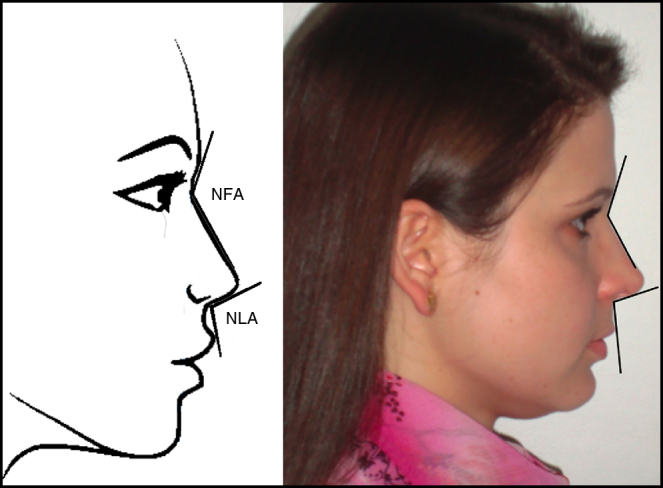
Lateral view: nasofrontal angle (NFA), nasolabial angle (NLA). Figure on the left: schematic model. Figure on the right: one of the volunteers in the study.

The obtained data was entered in an Excel spreadsheet. The measurements of the volunteers were compared between genders and with ideal measurements according to the literature described by Powell and Humphreys.^23^

The *alar distance–length ratio* is calculated by dividing the alar distance by the length of the nose (from nasion to pronasion). The pronasion, also called tip or tip defining point, is the point of projection of the most anterior nasal tip. The alar distance–length ratio of the ideal Caucasian nose is 0.7.^23^, ^24^

The *projection of the nasal tip* is the distance that the nasal tip protrudes from the face.^24^ In this study, Goode's method was used (Fig. 3). A vertical line is drawn from the nasion to the alar sulcus. Then, a horizontal line is drawn to the pronasion, perpendicular to this line. The connection between these two lines is called the alar point. The distance from the nasion to the pronasion was then measured. The ratio is obtained by dividing the distance from the alar point to the pronasion by the distance from the nasion to the pronasion. It is postulated that Goode's ratio ranges from 0.55 to 0.6.^23^

The *nasolabial angle* has its vertex at the subnasal point it is the angular inclination of the columella at the point where it meets the upper lip. A nasolabial angle ranging from 90° to 120° is considered ideal.^23^

The *nasofrontal angle* is obtained by tracing a line tangent to the glabella through the nasion that intersects with a line drawn tangent to the nasal dorsum. The ideal measure varies between 115° and 130°.^23^

The *alar-intercanthal distance ratio* is calculated by dividing the alar distance by the intercanthal distance. It is postulated that these measures are equivalent, therefore the ratio equal to 1 is considered ideal.^23^

According to the nature of the analyzed data, the statistical treatment that was considered appropriate was performed. For the planning of the study, the nasolabial angle was considered as the main variable. For this, a sample size was established, aiming to estimate the mean value of this variable, with an acceptable relative error of 2%, with a confidence level of 95%. To compare the anthropometric values obtained between genders, the mean standard deviation and Student's *t* test were performed. To compare the studied group with the esthetic values considered ideal, the statistical analysis included the mean standard deviation, 95% confidence interval and Two-tailed t test for each index. This was done by comparing the mean value of the sample with a fixed mean value of the ideal; the null hypothesis at each time was considered as no difference between the 2 groups. When the ideal value proposed in the literature was provided between two values, such as the nasolabial angle of 90–120°, the mean value was adopted (105°). For both tests, the *p*-value lower than 0.05 was considered to be statistically significant.

One hundred volunteers were included, 37 males and 63 females. The minimum age was 18 years and the maximum 54 years, with an average of 25.6 years. The mean age of men was 22.5 years, and of women 27.6 years.

## Results

The results of the anthropometric analysis obtained among the Caucasian volunteers of the city of Curitiba, and their comparison with the esthetic ideals are shown in Table 1. The mean values obtained in the population of Curitiba were: nasolabial angle of 105.41°; nasofrontal angle of 137.13; Goode's ratio of 0.63; alar width/length ratio of 0.85; alar/intercanthal distance ratio of 1.15. Only 6% of the population sample had an intercanthal distance equal to the alar distance, other 6% showed a greater intercanthal distance compared to the alar, while the great majority (88%) had a greater alar distance compared to the intercanthal. The alar distance was significantly greater than the intercanthal distance (*p* < 0.001). Comparing the results obtained in the population studied and those presented in the literature, except for the nasolabial angle, the population anthropometric measurements were statistically different and larger than the esthetic ideal (Table 1).

**Table 1:** Statistical analysis of nasal proportions obtained in Caucasians in the city of Curitiba and comparison with esthetic ideals.

Variable	Ideal value	Caucasians from Curitiba			*p*-Value
		Mean	SD	95% Confidence Interval	
Nasolabial angle,°	*105.0*	105.41	10.66	103.34–107.52	0.70
Nasofrontal angle,°	125.0	137.13	7.98	135.57–138.69	<0.001
Goode's ratio	0.58	0.63	0.05	0.62–0.64	<0.001
Alar width/length ratio	0.7	0.85	0.18	0.81–0.88	<0.001
alar/intercanthal distance ratio	1.0	1.15	0.1	1.13–1.17	<0.001

*Note*: Difference between means (normal distribution); level of significance <0.05.

*p* < 0.05.

The statistical analysis comparing the results obtained in the population sample studied between the genders is shown in Table 2. Only the nasofrontal angle (*p* = 0.0008) and Goode's ratio (*p* = 0.032) presented a significant difference between the genders. Men presented smaller nasofrontal angle and larger Goode's ratio than women (Table 2).

**Table 2:** Comparison between genders of nasal measurements obtained in the sample of the population studied.

Variable	Gender	*N*	Mean	SD	*p*-Value
Nasolabial angle,°	Male	37	107.75	9.82	
	Female	63	104.03	10.65	0.09

Nasofrontal angle,°	Male	37	133.71	6.43	
	Female	63	139.14	8.17	0.0008

Goode's ratio	Male	37	0.64	0.04	
	Female	63	0.62	0.06	0.032

Alar/intercanthal distance ratio	Male	37	1.17	0.09	
	Female	63	1.13	0.11	0.11

Alar distance/length ratio	Male	37	0.85	0.11	
	Female	63	0.86	0.12	0.61

Level of significance *p* < 0.05.

## Discussion

The plastic surgeon Leslie Farkas, dissatisfied with the determination of craniofacial morphology based solely on visual assessment, extensively studied human craniofacial measures and is considered the pioneer of modern craniofacial anthropometry.^25^, ^26^

In a study of rhinoplasty candidates and volunteers, although more than 89% of participants objectively had some asymmetric facial measure, 3 medical evaluators visually detected asymmetries in only 54% of volunteers and in 59% of rhinoplasty candidates, demonstrating the superiority of anthropometric measurements compared to the visual assessment.^27^

There are different methods of objective evaluation of nasal and facial measures, and they can be divided into anthropometric analysis and cephalometric evaluation. The anthropometric evaluation considers the points in the soft tissues, and can be divided into direct, when done directly in the patient, or indirect, through photographs, also called photogrammetry. The cephalometric study considers bone and soft tissue points through the use of standardized radiographs and photographs.^4^, ^24^, ^28^ In this study, nasal measurements were studied through indirect anthropometric evaluation, photogrammetry.

The use of photogrammetry has the advantage of high availability and documentation.^29^ However, as with direct anthropometric analysis, the quality of the results depends on the protocol for standardization.^1^, ^30^ There are differences between direct and indirect anthropometry measures, caused by photographic distortion, and it is advisable that certain measurements are obtained at certain angles that give more reliable and accurate results. For example, subnasal - pronasion is considered in the photo of the profile.^31^

**Figure 4:**
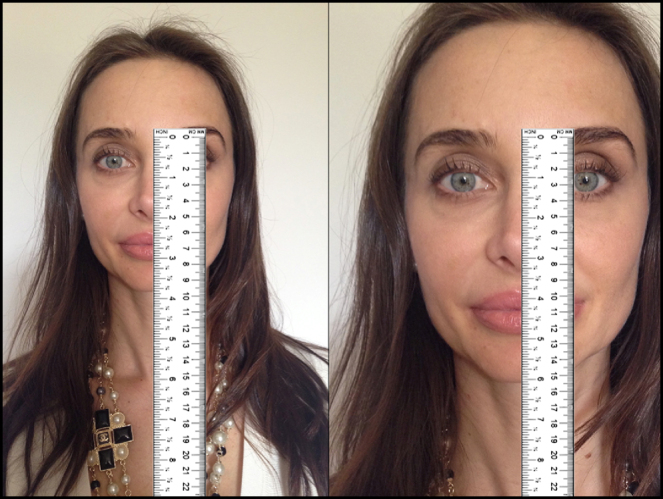
Example of the major problem of using absolute measures in photogrammetry. The same patient, therefore the same nose, but photos in different sizes, creating the illusion that the nose on the photo on the right is longer. This problem can be eliminated using ratios and angles between the primary measurements. Model: Fabiana Maros.

The great problem with photogrammetry is that even with a standardization protocol, the size of the image can vary a few millimeters from one photograph to another. Consequently, it is difficult to accurately measure distances between predetermined facial points in photographs. This problem can be eliminated using ratios and angles between the primary measures. In addition, the use of ratios and angles allows a certain flexibility in image size.^14^ Fig. 4 exemplifies the above, in an extreme but representative manner. Therefore, the present study evaluated only ratios and angles.

In this study, the values considered esthetic ideals of the nose were based on the descriptions by Powell and Humphreys,^23^ for its simplicity, generality and frequency with which it is cited and used in the literature. In addition, the values they described are usually consistent with most of the literature on this subject.^3^, ^4^, ^6^, ^17^, ^24^, ^32^, ^33^, ^34^

Comparing the female to the male sample, only the nasofrontal angle (*p* = 0.0008) and Goode's ratio (*p* = 0.032) showed a significant difference between the genders. Men had a sharper nasofrontal angle and a more projected nasal tip. In fact, Powell and Humphreys suggested that the nasal tip projection should be higher in men than in women.^23^

The currently used ideal esthetic pattern requires an equivalence between the intercanthal distance and the alar width. Since the alar/intercanthal distance ratio should result in 1, and the ratio found in 88% of the volunteers in this study was greater than 1, it is possible to state that the alar distance is greater than the intercanthal distance in the vast majority of the sample (*p* < 0.001). The Scottish population anthropometric study by Leong and White showed similar results. Among the 100 Scottish volunteers, 90% had an alar width greater than the intercanthal distance.^24^ This suggests that the nasal surgeon should be aware of techniques to reduce the width of the nasal base. On the other hand, the surgeon should also be aware that the alar width that is greater than the intercanthal distance is a population characteristic, and that in this case the patient may be accustomed to this characteristic and wishes to maintain this nasal aspect, or only minimize it.

Numerous authors cited by Powell and Humphreys mention that the nasolabial angle should ideally be between 90° and 120°, but there are authors who suggest that this angle be maintained between 90° and 105°. However, it is a consensus that male subjects should have this angle tending more toward the more acute value, while females tend more toward the obtuse one, suggesting a greater rotation of the nasal tip in women.^1^ In the population studied in this research, the average was 105.41° with no statistical significance between genders and between our population and the ideal form. This shows that the population sample has a rotation considered esthetically ideal.

The mean nasofrontal angle in the sample was statistically 12° larger, therefore more obtuse, than the average suggested by esthetic ideals. An obtuse nasofrontal angle may make a nose look longer, since the nasal root looks higher or poorly defined. This may be an important point for the learning of residents or less experienced surgeons to prevent diagnostic errors, which can easily occur in the population studied.

The width–length ratio was significantly higher (0.85) than the ideal (0.7), demonstrating that the population's noses have a broader base or a shorter nasal dorsum. Once 88% of the sample have an alar width greater than the intercanthal distance (*p* < 0.001), the broad base probably should contribute more to the high ratio than the dorsum length.

The projection of the nasal tip is measured for a reason. There are different ways of evaluating this ratio. The three evaluation methods that are most used in the literature are those by Simons, Baum and Goode. Simons described the measurement of the projection of the nasal tip in relation to the length of the upper lip.^35^ Although this is the simplest and most practical method of evaluation, there is an enormous variability in the length of the upper lip.^34^ Other methods commonly used in studies are Baum's method and Goode's method. The Baum's ratio is obtained by dividing the nasal length (nasion–subnasal) by the length of a perpendicular line of the pronasion to a vertical line that joins the pronasion to the subnasal. The nasal projection is the denominator of this ratio, so high ratios do not mean high projections, which can become confusing. Based on what was stated above, Goode's method was used in this study to analyze nasal tip projection.

The population sample had a more projected nasal tip than the ideal one (*p* < 0.001) (0.58–0.63). To check this information, statistical analysis was calculated by comparing the ratio obtained of the sample by Goode's method with the highest value of the ratio range (0.6 instead of the mean 0.58). The nose of the sample remained statistically more projected than the ideal. Other authors also found a larger nasal tip projection in the Scottish Caucasian population.^24^ However, these authors did not perform a statistical analysis. Another study found a higher Goode's ratio in 20 patients who were candidates for rhinoplasty (mean 0.65), but with the use of software that manipulates images to simulate the surgical outcome. Patients preferred a tip projection similar to that recommended by the ideal one.^33^

Interestingly, although the population sample from Curitiba shows a higher nasal projection than the ideal, studies show that the surgical technique performed in rhinoplasties in Curitiba does not aim to reduce the projection of the nasal tip.^20^, ^36^ On the contrary, in the article published in 2016, the authors aimed to increase the projection of the nasal tip in rhinoplasties.^36^ Is this population accustomed to a greater projection of the nasal tip? Are other nasal morphological factors more important for this population than nasal tip projection in rhinoplasty satisfaction? These doubts originated with the present study are extremely important, because the projection of the nasal tip is technically difficult, and can interfere in other nasal aspects, including the increase of the width of the alar base, and the reduction of air passage.^17^ A future study comparing rhinoplasty candidates with population measures can clarify these questions.

It is crucial to emphasize the importance of population studies. Knowing the nasal measurements of the population is useful to the surgeon because it can prevent diagnostic errors, such as the obtuse nasofrontal angle that can simulate a long nose. In addition, it may allow the surgeon to know which surgical techniques he/she should master. For example, in the studied population the surgeon should have mastery of techniques that reduce the nasal base. On the other hand, the surgeon is aware that the broad nasal base is a characteristic of this population, to which the patient may be accustomed to, and that he/she does not wish to change, or wishes only to change more slightly than the ideal recommends. Therefore, if you are looking for a natural result with rhinoplasty, some of these features can be maintained or just minimized. Finally, it makes it possible to discourage patients from requiring unnecessary interventions, and to preserve the innate nasal characteristics of the population to which they belong to.

Because the current nasal esthetics patterns do not consider population measures nor demographic and cultural aspects of the patient, which are considered relevant for the improvement of the patient's psychological well-being with rhinoplasty,^18^, ^19^ a discrepancy may occur between what the surgeon considers the patient's desire and what the patient really wants in a surgical outcome. Consequently, there is a greater chance of dissatisfaction with rhinoplasty.^24^

Through a validated questionnaire to evaluate satisfaction regarding facial appearance and quality of life after rhinoplasty, the literature showed that there was difference in satisfaction in both of them, according to the patient's demographic aspects. Female patients showed statistically significant improvement in facial appearance satisfaction and quality of life, while men were satisfied only with the facial appearance. Caucasian patients demonstrated a statistically significant improvement in satisfaction regarding quality of life and facial appearance while non-Caucasians showed no increase.^19^

Although the population sample of Curitiba, due to the high prevalence of Caucasians, is theoretically one of the Brazilian population samples that most resembles the esthetic nasal patterns, except for the nasolabial angle, the population sample differed from the ideal ones. This does not mean that the nose of the population is imperfect, only that the population differs from the classic ideal. This consideration is extremely important when counseling the patient before plastic surgery because it is less important to base on esthetic ideals than to consider the nose and facial proportions of the patient individually and as a whole. The measurements obtained demonstrate the necessity and importance of population studies for reference, evaluation and preoperative counseling before nasal plastic surgery.

## Conclusion

The population sample of Curitiba differs statistically from current ideal esthetic nasal patterns, with the exception of the nasolabial angle.

In the comparison between genders, in males we found a more acute nasofrontal angle and a more projected nasal tip.

## Conflicts of interest

The authors declare no conflicts of interest.
